# Successful treatment of generalized myasthenia gravis with telitacicept: a Chinese case series and literature review

**DOI:** 10.3389/fneur.2025.1501500

**Published:** 2025-01-31

**Authors:** Xiaodong Song, Yang He, Hong Jiang, Yao Yu, Yue Sun, Zhaoxu Zhang

**Affiliations:** ^1^Department of Neurology, Peking University People’s Hospital, Beijing, China; ^2^Department of Neurology, The First Affiliated Hospital of Chongqing Medical University, Chongqing, China

**Keywords:** generalized myasthenia gravis, telitacicept, B lymphocyte stimulator, a proliferation-inducing ligand, treatment

## Abstract

**Background:**

Despite existing treatments of generalized myasthenia gravis (gMG), there remains a need for more effective therapies with fewer side effects. Telitacicept, targeting B lymphocyte stimulator (BLyS) and a proliferation-inducing ligand (APRIL), emerges as a potential novel therapy for gMG.

**Case presentation:**

In our study, four patients with gMG to standard treatments underwent an 8-week course of telitacicept monotherapy. Post-treatment, all patients exhibited satisfactory improvements. The Myasthenia Gravis Foundation of America Quantitative Myasthenia Gravis (MGFA-QMG) scores, 15-item Myasthenia Gravis Quality of Life (MGQOL-15) scores, and MG-associated Activities of Daily Living (MG-ADL) scores showed a marked reduction, indicating decreased disease severity and enhanced quality of life. Additionally, immunological assessments revealed a decrease in CD19^+^B lymphocyte counts and acetylcholine receptor (AChR) antibodies. Only one patient reported a mild, transient injection reaction.

**Conclusion:**

Favorable clinical improvement and mild adverse events for gMG in treated with telitacicept were observed. However, larger-scale and longer-term studies are necessary to confirm these results and fully establish the role of telitacicept in the treatment of gMG.

## 1 Introduction

Myasthenia gravis (MG), an severe autoimmune neuromuscular disorder, predominantly manifests through skeletal muscle weakness and fatigability, impacting approximately 12.4 individuals per 100,000 population worldwide (1, 2). This condition is characterized by the production of pathogenic antibodies, most commonly targeting the acetylcholine receptors (AchR) or muscle-specific kinase (MuSK) at the neuromuscular junction, leading to impaired neuromuscular transmission (3). Initial therapeutic strategies for MG typically involve anticholinesterase inhibitors, such as pyridostigmine, which provide symptomatic relief but do not alter the underlying disease course (4). For rapid, albeit short-term improvement in clinical status, intravenous immunoglobulin (IVIG) and plasma exchange (PE) are frequently employed (5, 6). The long-term management of MG often necessitates the use of corticosteroids and other immunosuppressive agents like azathioprine, mycophenolate mofetil, and methotrexate (4). However, these traditional treatments, while effective in many cases, can lead to significant side effects and may not suffice for all patients (7, 8). Particularly challenging is the management of refractory generalized myasthenia gravis (gMG), which affects an estimated 10–15% of MG patients (9–12). These clinical challenges are compounded by increased healthcare utilization and a diminished quality of life for patients (13).

Telitacicept, a novel biologic agent, heralds a new era in the management of autoimmune diseases. This recombinant fusion protein, comprising the transmembrane activator and CAML interactor (TACI) receptor fused with the fragment crystallizable (Fc) region of human immunoglobulin G (IgG), operates by neutralizing the activities of B lymphocyte stimulator (BLyS) and a proliferation-inducing ligand (APRIL) (14). This dual blockade results in a significant reduction of circulating B cells, pro-inflammatory cytokines, and IgG levels, thereby modulating the immune response. In the realm of rheumatic diseases, telitacicept has demonstrated efficacy and safety in conditions such as systemic lupus erythematosus (SLE), neuromyelitis optica spectrum disorders, Sjogren’s syndrome, and autoimmune nephropathy (15–18). Its mechanism of action, targeting key pathways in the pathogenesis of autoimmune disorders, offers a unique therapeutic approach by concurrently inhibiting the proliferation and maturation of B and T lymphocytes (19). Despite these advances, the precise therapeutic efficacy of telitacicept in gMG remains an area of ongoing research and clinical interest, underscoring the need for further studies to elucidate its role in the treatment paradigm of this complex autoimmune disorder.

Therefore, in this context, we aim to share our experience of telitacicept in treating gMG in four cases. This report may provide a novel approach for patient management, contributing valuable insights to the evolving landscape of gMG treatment strategies.

## 2 Case presentation

Patient 1, a 67-year-old female, presented with a complex, progressive course of myasthenia gravis, initially diagnosed 5 years ago. Her initial symptomatology included right eyelid ptosis, which manifested acutely and displayed diurnal fluctuations, notably more pronounced in the evening and ameliorating with rest. This ocular manifestation led to a diagnosis of ocular MG, for which she commenced treatment with pyridostigmine, dosed at 60 mg thrice daily. Approximately 3 years after the initial diagnosis, the patient’s condition evolved, marked by the emergence of limb weakness and left eyelid ptosis, substantially impairing her vision. Despite therapeutic adjustments to the pyridostigmine regimen, these symptoms persisted. This refractory response necessitated the addition of oral prednisone (10 mg daily) and tacrolimus (3 mg daily), resulting in a gradual improvement of both limb weakness and eyelid ptosis. However, 4 years post-diagnosis, the patient experienced an exacerbation of her symptoms. This relapse was characterized by an intensified limb weakness, notably impacting her ability to perform tasks such as climbing stairs and lifting heavy objects. Concurrently, she developed new symptoms including hoarseness and dysphagia, predominantly with solid foods. These clinical manifestations prompted her admission to our hospital, where she was re-evaluated and diagnosed with gMG.

Upon admission, assessments revealed a Myasthenia Gravis Foundation of America Quantitative Myasthenia Gravis (MGFA-QMG) score of 19, indicative of moderate disease severity. Complementary to this, her 15-item Myasthenia Gravis Quality of Life (MGQOL-15) and MG-associated Activities of Daily Living (MG-ADL) scores were 20 and 15, respectively, reflecting a considerable impact on her quality of life and daily functioning. Diagnostic confirmation was obtained through an Enzyme-Linked Immunosorbent Assay (ELISA), which identified the presence of AChR antibodies in her blood, corroborating the diagnosis of MG. In light of her clinical presentation and laboratory findings, a decision was made to initiate treatment with telitacicept. This therapeutic transition involved discontinuing her existing regimen of tacrolimus and prednisone. The response to telitacicept was promising. Merely 2 weeks post-initiation, marked improvement in clinical symptoms was noted, particularly in swallowing difficulties and hoarseness. Objective measures supported this clinical observation; her MGFA-QMG, MGQOL-15, and MG-ADL scores decreased by 6, 4, and 3 points, respectively, The patient’s improvement continued positively; by the eighth injection, which was administered 7 weeks after the initial dose, further amelioration of symptoms was evident. The scores, as compared to the baseline, were marked reduced (MGFA-QMG by 17 points, MGQOL-15 by 20 points, and MG-ADL by 15 points). In response to these favorable outcomes, it was decided to discontinue the medication, transitioning the patient to a monitoring phase. During the one-month clinical follow-up, the patient’s condition remained stable, affirming the efficacy of the telitacicept treatment in managing her gMG symptoms.

**Figure 1:**
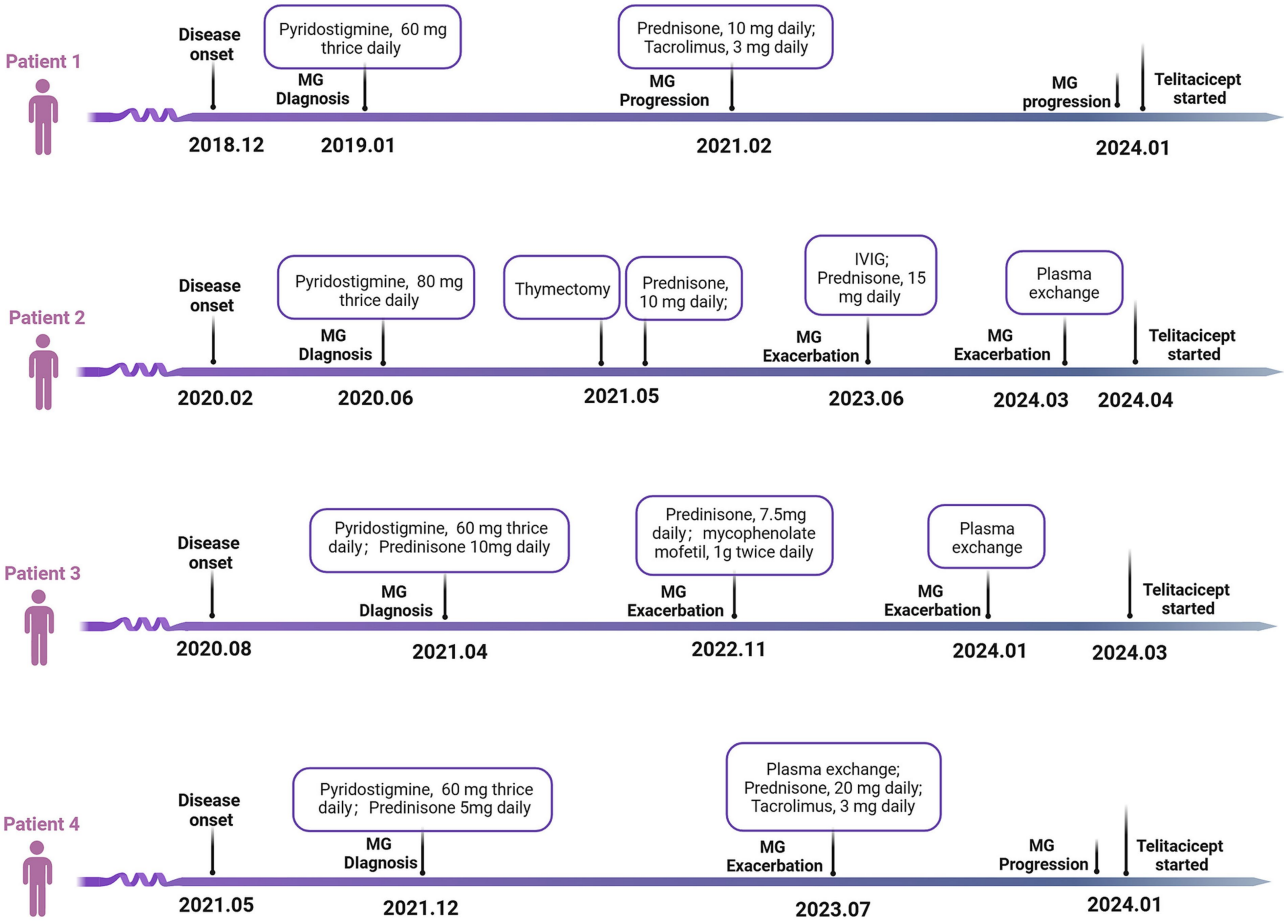
Medical history of the four refractory gMG patients. gMG, generalized myasthenia gravis; IVIG, intravenous immunoglobulin.

**Figure 2:**
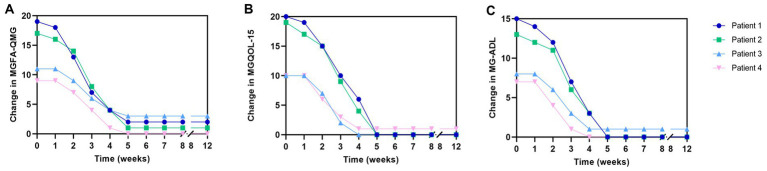
Clinical scale changes following telitacicept treatment from basline to week 12. **(A)** Change from baseline to week 12 in MGFA-QMG. **(B)** Change from baseline to week 12 in MGQOL-15. **(C)** Change from baseline to week 12 in MG-ADL. MGFA-QMG, Myasthenia Gravis Foundation of America Quantitative Myasthenia Gravis Score; MGQOL-15, 15-item Myasthenia Gravis Quality of Life Scale; MG-ADL, MG-associated Activities of Daily Living score.

Concurrently, three other patients, each with a diagnosis of gMG, were treated with telitacicept monotherapy. The clinical characteristics and medical history of these four individuals are comprehensively summarized in Table 1 and Figure 1. Echoing the experience of patient 1, patients 2, 3, and 4 exhibited notable improvements in their gMG clinical scores within the initial 2–4 weeks of starting telitacicept treatment. This rapid clinical response was characterized by a marked reduction in MGFA-QMG, MGQOL-15, and MG-ADL scores, reaching a peak around the fifth week of therapy. Notably, this enhanced clinical state was sustained, as demonstrated by the stable scores observed in these patients (Figure 2). Upon the conclusion of the 8-week telitacicept course, treatment was discontinued for all patients. Interestingly, during the one-month follow-up period post-telitacicept withdrawal, their gMG clinical scores remained unchanged, suggesting a sustained therapeutic effect of the drug (Figure 2). Patient 3 experienced a transient injection reaction, which manifested as a mild flu-like reaction with symptoms of fever, sore throat, and headache. These symptoms were self-limiting and resolved spontaneously without necessitating discontinuation of telitacicept. However, no other adverse event was observed, underscoring the tolerability of this therapeutic agent in the management of gMG.

**Table 1:** Patient characteristics.

Patient no.	Sex	Age of onset	Antibody	Disease duration	Thymoma type	Immunosupressant before telitacicept	Dose of telitacicept	Baseline MGFA-QMG	Baseline MG-QOL15	Baseline MG-ADL
1	F	62	AchR	60 months	None	Prednisone, tacrolimus	160 mg/week	19	20	15
2	F	61	AchR	49 months	AB	Thymectomy, prednisone	160 mg/week	17	19	13
3	M	69	AchR	42 months	None	Prednisone, mycophenolate mofetil	160 mg/week	11	10	8
4	F	59	AchR	32 months	None	Prednisone, tacrolimus	160 mg/week	9	10	7

M, male; F, female; AChR, acetylcholine receptor; AB, Thymoma AB type; MGFA-QMG, Myasthenia Gravis Foundation of America Quantitative Myasthenia Gravis Score; MGQOL-15, 15-item Myasthenia Gravis Quality of Life Scale; MG-ADL, MG-associated Activities of Daily Living score.

**Figure 3:**
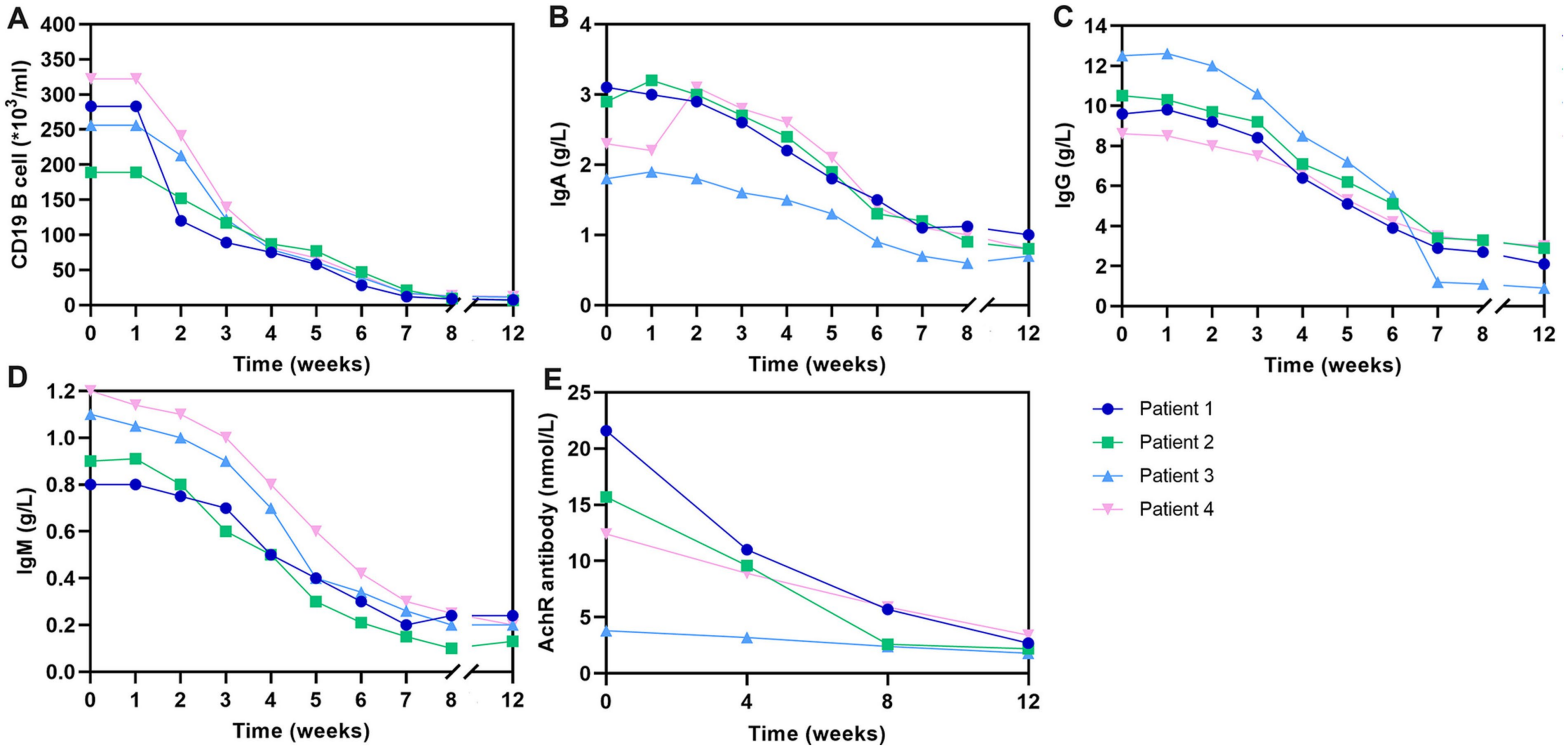
Change in CD19^+^ B cells and antibody levels over the 12-week study period. **(A)** Changes in CD19^+^ B-cell counts over the study period. **(B)** Changes in IgA levels over the study period. **(C)** Changes in IgG levels over the study period. **(D)** Changes in IgG levels over the study period. **(E)** Changes in AChR levels over the study period. AChR, acetylcholine receptor; IgA, immunoglobulin A; IgG, immunoglobulin G; IgM, immunoglobulin M.

In the course of routine clinical management, we conducted dynamic monitoring of key immunological parameters in these patients, including CD19^+^B lymphocyte counts, and levels of IgA, IgM, IgG, and AChR antibodies. These assessments, integral to our therapeutic protocol, were performed following the initiation of telitacicept treatment. Using flow cytometry, we observed a marked decrease in CD19^+^B lymphocytes as early as one-week post-initial telitacicept administration. This trend of declining CD19^+^B cell counts was consistent throughout the treatment duration, up to week 8 (Figure 3A). Parallel to the changes in CD19^+^B cells, we noted a weekly decrement in the concentrations of IgA, IgM, and IgG antibodies in the peripheral blood of all four patients. This pattern is indicative of the immunomodulatory effect of telitacicept (Figures 3B–D). Additionally, the levels of AChR antibodies, a pivotal marker in the management of GMG, were evaluated at four-week intervals. Consistent with the therapeutic goal, there was an overall downward trajectory in AChR antibody levels throughout the treatment period, further supporting the clinical effectiveness of telitacicept in these patients (Figure 3E).

## 3 Discussion

In this case series, we explored the clinical efficacy of telitacicept in the treatment of gMG, a novel approach in the therapeutic landscape of this autoimmune disorder. Our study involved four patients, each with distinct manifestations of gMG, all of whom showed significant improvements following an 8-week course of telitacicept monotherapy. This was reflected in marked reductions in gMG clinical scores, including the MGFA-QMG score, and improvements in patients’ quality of lives, as measured by the MGQOL-15 and MG-ADL scores. These findings suggest that telitacicept not only reduces disease severity but also improves functional capacity, making it a promising alternative to traditional corticosteroid therapy, which is often associated with adverse side effects (7, 8).

Our findings align with three published case reports describing treatment with telitacicept. For instance, a 75-year-old female with gMG showed significant improvement in both clinical symptoms and MG scores following treatment with telitacicept (20). Similarly, two other patients—one with concurrent gMG and rheumatoid arthritis, and another with an eight-year history of gMG—also experienced substantial symptom reductions after receiving the same therapy (21). These observations are further supported by a recent phase II randomized controlled trial, which confirmed that telitacicept effectively and rapidly alleviates gMG symptoms over a six-month period, while maintaining a favorable safety profile (22).

The safety profile of telitacicept observed in our study was favorable, with only one patient experiencing a mild, transient injection site reaction. Immunologically, telitacicept led to a decrease in CD19 ± B lymphocyte counts and reduced levels of IgA, IgM, IgG, and AChR antibodies. These changes align with the drug’s mechanism of action, which involves neutralizing BLyS and APRIL to suppress autoimmune humoral immunity and lower autoimmune antibody levels. However, the reduction in IgG levels observed in our study raises concerns about potential infection risks. To mitigate this risk, we recommend regular immune function monitoring and vaccination before starting treatment, particularly for influenza and pneumonia.

Recent advances in the treatment of gMG have provided more targeted therapeutic options. Agents like complement inhibitors and neonatal Fc receptor blockers offer promising approaches due to their specificity in targeting disease pathways (5, 23, 24). B-cell depletion therapies, such as rituximab, have demonstrated efficacy in refractory gMG (25–27), though its effect on reducing steroid dependence in mild-to-moderate cases remains limited (28). Targeting key cytokines like B-cell activating factor (BAFF or BLyS), which is crucial for B-cell survival, is another strategy gaining attention. While belimumab, a BAFF inhibitor, has shown efficacy in systemic lupus erythematosus (29), its benefit in gMG is uncertain, as demonstrated by a phase II trial (30). These developments underscore the complexity of gMG treatment and the need for continued exploration of more targeted therapies. Our findings suggest that telitacicept could be particularly valuable for gMG who have not responded to conventional therapies or are unable to tolerate their adverse effects. However, given the limitations of our study, including the small sample size and short follow-up, further research is essential to better define its role in clinical practice.

## 4 Conclusion

In conclusion, telitacicept demonstrates promising efficacy in treating gMG, improving both clinical scores and quality of life while maintaining a favorable safety profile in the short term. For clinicians, these findings provide valuable guidance when considering telitacicept as a treatment option, particularly for gMG patients who are unresponsive to or intolerant of traditional therapies. However, further research with larger cohorts and longer follow-up is essential to establish the long-term safety and efficacy of telitacicept in gMG patients.

## Data Availability

The datasets presented in this article are not readily available because of ethical and privacy restrictions. Requests to access the datasets should be directed to the corresponding author.
